# Assessing the Host Range of *Ophraella communa* for the Biological Control of *Ambrosia artemisiifolia* in France

**DOI:** 10.3390/plants13223240

**Published:** 2024-11-19

**Authors:** Zoé Rousset, Alberto Zamprogna, Coline C. Jaworski, Nicolas Desneux, Vincent Lesieur

**Affiliations:** 1 Université Côte d’Azur, INRAE, UMR ISA, 06000 Nice, France; coline.jaworski@inrae.fr; 2 CSIRO Health & Biosecurity European Laboratory, 34980 Montferrier sur Lez, France; alberto.zamprogna@csiro.au (A.Z.);

**Keywords:** common ragweed, behavior, choice, biocontrol, host specificity, preference–performance relationship, ragweed beetle specialization

## Abstract

*Ambrosia artemisiifolia* is a well-known invasive species in Europe, causing health issues with its extremely allergenic pollen and yield loss through competition in agriculture. One potential biological control agent is *Ophraella communa*, accidentally introduced in Europe in 2013. This species was discovered in France in 2023, but before planning further mass releases for biological control, it is necessary to assess its safety for agricultural crops and local plant biodiversity. Prior to its first detection in France, we conducted a host-range study of the beetle in a confined laboratory with no-choice and choice tests for 16 plant species, selected based on the centrifugal phylogenetic method. Results showed a restricted host range to the Heliantheae tribe and minimal risk to indigenous European plant species, with no larval survival and barely any eggs laid on these plants. Choice tests also showed a strong preference of *O. communa* for *A. artemisiifolia*. Our results combined with previous studies and observations in the field in other countries suggest a low risk to sunflower, *Helianthus annuus*, which is an important crop in France. This confirms that *O. communa* could be a low-risk biological control agent that can be used in classical biological control programs against *A. artemisiifolia* in France.

## 1. Introduction

Impacts of invasive alien species on biodiversity, agriculture, or human health are now very well documented and acknowledged by the scientific community. For example, invasive alien species account for 60 percent of documented global extinctions [1]. Likewise, Haubrock et al. (2021) estimated that biological invasions in Europe costed an average of €1.91 billion annually from 1960 to 2020, with costs increasing exponentially by ten-fold each decade. In 2017, the most costly invasive alien species in France was a plant species, *Ambrosia artemisiifolia* L. (Asteraceae: Heliantheae: Ambrosiinae), with a total estimated cost of US $551 million [2].

*Ambrosia artemisiifolia*, also known as common ragweed, is native to North America and was observed in France for the first time in the 1870s [3]. This annual monoecious weed develops in cultivated, semi-natural, or disturbed open areas [4,5]. Its highly allergenic pollen causes health issues such as rhinitis, conjunctivitis, tracheitis, and less frequently quite serious forms of asthma [6]. In Europe, an estimated 23.2 million inhabitants are ragweed-sensitized, leading to an annual economic cost of about €7.4 billion due to pollen allergies [7]. *Ambrosia artemisiifolia* can also cause severe yield loss in spring crops (soybean, sunflower, etc.), which can be up to 84% in soybean [8,9].

In the Milan area, Italy, the concentration of *A. artemisiifolia* pollen significantly decreased in 2013, which was then correlated with the accidental introduction of the ragweed leaf beetle, *Ophraella communa* LeSage, (Coleoptera: Chrysomelidae), discovered during the summer of 2013 [10]. This beetle, also native to North America, is an oligophagous insect that has a strong preference for plant species in the genus *Ambrosia*, especially for *A. artemisiifolia*, on which it completes its entire development cycle [11,12]. High densities of beetles can lead to complete defoliation and ultimately plant death [13,14]. In Europe, since its first observation in Italy in 2013, it has spread eastward, reaching Bucharest in Romania in 2023 [15,16]. *Ophraella communa* was also accidentally introduced in several Asian countries (China, Japan, and South Corea) [17,18], and since its introduction, the beetle is now widely used as a biological control agent in China where it is mass-reared and released in the environment to control *A. artemisiifolia* populations [13,19].

By contrast, west of the Alps, the beetle was absent until very recently; indeed, it was detected in several locations in southeast-central France (Auvergne Rhône Alpes region) in October 2023 [20]. Incidentally, an earlier study analyzing changes in airborne *A. artemisiifolia* pollen concentrations in northern Italy (2008–2012 and 2013–2015) suggested that the presence of *O. communa* in this area could help reducing *A. artemisiifolia* pollen concentration by 76% as well as related healthcare costs by 75–85% [21,22]. Likewise, at the European level, Schaffner et al. (2020) estimated that the use of *O. communa* against *A. artemisiifolia* would reduce the number of people affected by *A. artemisiifolia* pollen allergies by 2.3 million [7].

While selecting a biological control agent, it is important to identify its host range to evaluate the risk for non-target species [23]. Various parameters, including adult feeding, fecundity, oviposition site selection, larval feeding, and survival, are considered in host range evaluations, depending on the insect being tested [23]. The “centrifugal phylogenetic method” guides the selection of plant species to be tested, hypothesizing that species closely related to the target species are more likely to be attacked by the biological control agent than more distant ones [24,25].

Several studies assessing the host range of *O. communa* have been already carried out outside of its native range [26,27,28]. Kim and Lee (2019) reported eighteen potential host plant species, with six species in the genus *Ambrosia*, three in the genus *Xanthium*, and three in the genus *Helianthus*, including sunflower *Helianthus annuus* L. [11]. Although the beetle was rejected for introduction in Australia as a biological control agent due to its ability to complete its life cycle on sunflowers under laboratory conditions [29], later studies concluded that *O. communa* poses no threat to sunflowers [28,30,31]. However, all these risk assessments for sunflowers were performed in its native range or in China, with different cultivars than the cultivated ones in France. Sunflower is an important crop in France with 868,000 ha planted in 2023 [32]. Consequently, before using *O. communa* as a biological control agent against *A. artemisiifolia*, it is important to clearly assess the risk for sunflower production. Furthermore, the only study that assessed its host range within native European plant species found no evidence of substantial non-target effects that can threaten populations of such native plant species [26], but only a few plant species, primarily from the Helenieae tribe (especially several *Pentanema* species), were included in this study, and it seems important to fill in the gap.

In this context, our study aims to extend the risk assessment of *O. communa* for Europe by evaluating the beetle’s host range with more European plant species, both native and introduced, with a view toward classical biological control of *A. artemisiifolia* in France. No-choice tests were performed on 16 species, with 10 indigenous plants and six introduced plants (crops or weeds), including seven French sunflower cultivars—the potential main economical concern in the case of *O. communa.* Choice tests were also conducted on plant species supporting larval survival and/or plant species on which females laid eggs. In addition, we tested the preference performance relationship [33,34,35] to evaluate the specialization degree of *O. communa*.

## 2. Material and Methods

### 2.1. Host Plants Tested

Plant species were selected according to their phylogenetic relationships to the target plant as described in the centrifugal phylogenetic method [24]. A special focus was on species with economic (sunflower and other exotic species), and environmental (indigenous species) importance (Table 1).

*Ambrosia artemisiifolia* is part of the sub-tribe Ambrosiinae from the Heliantheae tribe. Few species of this tribe are naturally present in Europe. Two Ambrosiinae were selected—*Ambrosia trifida* L. and *Xanthium orientale* L. (both are exotic invasive weeds in France). From the Heliantheae tribe, three economically important species were chosen: the sunflower *H. annuus*, of which seven cultivars were selected; *Helianthus tuberosus* from the Helianthinea sub-tribe; *Cosmos sulphureus* L from the Coreopsidinae sub-tribe. Another Coreopsidinae, *Bidens cernua* L., which is indigenous in France, was tested. The seven sunflower cultivars selected were as follows: RGT BUFFALO and RGT AXELL from *RAGT Semences*; SY CELESTO from *Syngenta*; MAS 89HOCL from *Mas Seeds*; ES IDILLIC and ES VERONIKA from *LIDEA*; P64HE118 from *Pioneer Semences*.

The Helenieae tribe is the most closely related tribe to Heliantheae that occurs naturally in Europe [36]. Therefore, we selected several species within this tribe to obtain an overview of the risk to this related taxon. We chose *Pallenis spinosa* L., *Carpesium cernuum* L., *Pentanema bifrons* L., *Pentanema britannicum* L., and *Pentanema helveticum* Weber. Finally, we added some species from more distant tribes: *Artemisia molinieri* Quézel, *Matricaria chamomilla* L., *Erigeron sumatrensis* Retz, *Bellis perennis* L., and *Centaurea solstitialis* L. The relationships between the tested plant species to *A. artemisiifolia* are illustrated by a phylogenetic tree (Supplementary Materials, Figure S1).

### 2.2. Plant Production

The seeds of the different tested plants species were obtained from scientific institutes (botanical gardens or National Botanical Conservatoire (NBC)), private companies, or direct collections in the field (Supplementary Materials, Table S1). *A. artemisiifolia* seeds were collected in the French department Côte d’Or by colleagues from INRAE Dijon (UMR1347 Agroécologie). All seeds were kept in paper envelopes in an air-conditioned storage room.

To break dormancy, seeds of *A. artemisiifolia* were placed for two weeks at 4 °C in Petri dishes with watered vermiculite on a filter paper. Then, the seeds were transferred at 25 °C for germination and transplanted into 9 × 9 × 9.5 cm^3^ pots with standard horticultural soil (Neuhaus Humin Substrat N6; Klasmann-Deilmann GmbH, Geeste, Germany). All plants were grown the same way, with or without seed cold treatment, in a climatic chamber at 25 °C with a photoperiod of 16 h L: 8 h D.

### 2.3. Insect Rearing

The insects used in all experiments were sourced from a laboratory colony initiated with *O. communa* collected in Italy in August 2021 and north-west of Milan (45°34′14.4″ N ; 8°47′09.5″ E), during which several dozens of insects were collected. The insect population was maintained in the containment laboratory of the CSIRO European Laboratory at 23 °C with a photoperiod of 16 h L: 8 h D, a relative humidity of 60% during night, and with natural fluctuations during the rest of the day. Adults and larvae were kept separately in 40 × 40 × 60 cm^3^ screened cages with unlimited access to *A. artemisiifolia* plants. Eggs were collected every two days in the adult cages and placed in Petri dishes with humid filter paper for incubation. Freshly emerged larvae were used for the experiment. When larvae started to pupate, they were placed in 15 × 9.5 × 9.5 cm^3^ plastic boxes until adult emergence. Freshly emerged adults (<24 h) were used for the experiment.

### 2.4. No-Choice Tests

All tests were conducted in the same condition as insect rearing in the containment laboratory. Two different laboratory experiments were performed: (I) non-target plant species exposed to two couples (two females and two males) of *O. communa* adults; (II) non-target plant species exposed to ten larvae. Each treatment consisted of five replicates. For each replicate, we placed one plant of the non-target species in a screened cage (40 × 40 × 60 cm^3^). Due to space constraints in the containment laboratory, we spaced the experiment in different series, using *A. artemisiifolia* as control (five replicates of some non-target species always with five replicates of *A. artemisiifolia*). Plant size ranged from 10 cm to 40 cm depending on the plant species.

The experiment with adults lasted for 3 weeks to remain consistent with previous studies [29] or until they were all dead. Adults that died within 48 h after having been introduced were replaced to avoid biasing the results. We placed the adults at the bottom of the cage, and at the beginning of the experiment, the cages were inspected daily to record the preoviposition period (i.e., the number of days between female emergence and the beginning of egg laying). Then, every two or three days, eggs masses, total number of eggs, and viable eggs were counted and removed from the plant, and adult mortality was checked. Viable eggs were estimated visually. Viable eggs are typically yellow-orange, firm, smooth, and pyriform [12], while inviable eggs often differ in color and texture; they may turn brown and generally appear shriveled or soft as they deteriorate.

For larvae, they were deposited directly on the leaves of plants, and the tests lasted until all larvae had pupated or died. At the end of the experiment, pupae were weighed (microbalance accurate to 0.001 mg) and individualized in glass tubes with a vented lid until adult emergence. The tubes were inspected daily to record the emergence date. Adults were then sexed using sexual dimorphism as described by LeSage (1986) [12].

### 2.5. Choice Tests

Only plant species that enabled larval development to adulthood and/or on which females laid eggs in no-choice tests were included in the choice tests. Consequently, the plant species tested were *A. trifida*, *X. oriental*, *C. cernuum*, *P. helveticum*, and *H. annuus.*

Choice tests included eight replicates and consisted of introducing one plant of the non-target species with one *A. artemisiifolia* plant in the same cage (40 × 40 × 60 cm^3^), exposed to two couples of *O. communa* adults. Because of the abnormal behavior of the insects (no egg laid, neither on *A. artemisiifolia* or the plant tested), we had to remove one replicate for *C. cernuum* and one for *P. helveticum* (seven replicates for these two plant species). The experiment lasted three weeks. If adults died, they were replaced with new ones, as we were looking at the adult preference. Every two or three days, we removed and counted egg masses, total number of eggs, and egg viability from each plant in the cage. We also measured herbivory damage in both plants once a week. Damage *D* was visually estimated as the percentage of leaf area damage (averaged on ten damaged leaves), and herbivory was calculated with the following formula: *Herbivory = N_d/_N_t_ × D,* with *N_d_* as the number of damaged leaves and *N_t_* the total number of leaves.

### 2.6. Data Analysis

All statistical analyses were conducted using R Core Team (2023) version 4.3.1 (RStudio 2023.06.1) [37] and using the packages *readr* [38], *tidyverse* [39], and *rstatix* [40]. Figures were produced with *ggplot2* and *ggpubr* [41]. Normality and homoscedasticity were checked with the package *DHARMa* [42].

No-choice data were analyzed by assessing the effect of plant species on the total number of egg masses, number of viable eggs, adult mortality, length of the preoviposition period, larval survival, and larval development time (from first instar to adulthood). As most of the data did not follow normality and/or homoscedasticity rules, we used Kruskal–Wallis’ tests, followed by Wilcoxon tests when significant. The *p*-values were adjusted with the method by Benjamini and Hochberg (1995) [43]. Adult survival probability was analyzed with a cox regression with R package *survival* [44].

As the number of larvae surviving in the no-choice test was low for most of the non-target plant species, we could only analyze pupal weight data from *H. annuus,* by grouping the different cultivars together, and from *A. artemisiifolia*, with data from the different series of tests. As these data followed a normal distribution and the homoscedasticity rule, we analyzed the effect of the plant (*H. annuus* versus *A. artemisiifolia*) and the effect of the sex on pupal weight with a generalized linear model (GLM) with a normal distribution.

For assessing the preference–performance hypothesis [33,34,35], we tested for correlation between the number of eggs laid by females and the larval survival rate on plant species tested (*cor.test* function from *rstatix* package [40]). We used Kendall’s method, as the data did not follow a normal distribution.

For choice tests, we assessed the adult host plant choice between *A. artemisiifolia* and the alternative plant species by comparing the number of egg masses and the number of viable eggs laid on each plant and the herbivory damage with Wilcoxon tests for independent samples (Wilcoxon rank sum test with continuity correction), as the normality and/or homoscedasticity rules were not respected.

To estimate the risk of non-target attacks by *Ophraella communa*, we calculated the “combine score” as described by Paynter et al. (2015), which consists of multiplying the relative performance measures (e.g., the performance of *O. communa* on a test plant, divided by the same performance measure on *A. artemisiifolia*) for oviposition and starvation tests together [45]. We also calculated the Preference and Performance Index (PPI), which takes the square root of relative choice oviposition multiplied by relative larval development, as described by Grevstad et al. (2021) [46].

## 3. Results

### 3.1. No-Choice Tests

**Figure 1:**
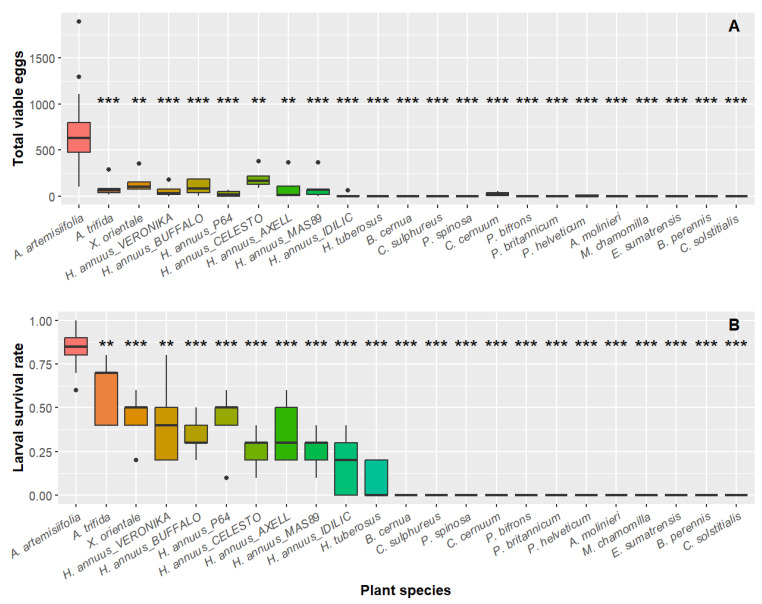
Results of the no-choice experiment with *Ophraella communa*: (**A**) number of viable eggs laid by females and (**B**) larval survival rate on the different plant species. Plant species are ordered according to their phylogenetic relatedness to *Ambrosia artemisiifolia*. Asterisks show the degree of significance between the plant species tested and the control plant, *A. artemisiifolia*, according to the Wilcoxon test, with *p*-value adjustment using the method by Benjamini and Hochberg (1995). ** *p* < 0.01; *** *p* < 0.001.

The plant species exposed to *O. communa* adults in no-choice tests had a significant effect on the number of egg masses laid (*H* = 122; *df* = 22; *p* < 0.001), the number of viable eggs (*H* = 124; *df* = 22; *p* < 0.001), the preoviposition period (*H* = 40.4; *df* = 12; *p* < 0.001), the larval survival rate (*H* = 126; *df* = 22; *p* < 0.001), and the larval development time (*H* = 40.9; *df* = 10; *p* < 0.001) (Table 2, Figure 1). There was no difference in the sex ratio of emerging adults between plant species that allowed larval development to adulthood (*H* = 11.8; *df* = 10; *p* = 0.30).

The Wilcoxon test showed that the number of egg masses, the number of viable eggs, and the larval development to adulthood rate were lower for all non-target plant species compared to *A. artemisiifolia* (Figure 1, Supplementary Table S2). Eggs were obtained from *A. trifida*, *X. orientale*, all cultivars of *H. annuus*, *C. cernuum*, and *P. helveticum*, while no eggs were laid on the remaining 11 tested species (65% of species tested). Almost all the species on which females laid eggs supported larval survival. For *H. tuberosus*, in the no-choice test with larvae, 8% (±1) of the larvae survived, despite females not laying any eggs in the no-choice with adults. For the remaining 12 tested plant species (71% of the species evaluated; see Figure 1, Supplementary Table S2), all larvae died, including *C. cernuum* and *P. helveticum*, on which females laid eggs during the no-choice tests with adults. Concerning the preoviposition period and larval development time, we performed a Wilcoxon test only between *A. artemisiifolia* and plant species for which oviposition was observed and/or for which at least one larva had survived in more than one replicate (Table 2). The preoviposition period tended to be longer on non-target plant species, but this trend was only significant for *A. trifida* (*W =* 30.0, *p* = 0.037), *X. orientale* (*W* = 33.0, *p* = 0.040), *H. annuus* cultivars AXELL (*W* = 1.5, *p* = 0.003), BUFFALO (*W* = 23.5, *p* = 0.026), VERONIKA (*W* = 26.5, *p* = 0.031), MAS89 (*W* = 34.0, *p* = 0.040), and *C. cernuum* (*W* = 0.0, *p* = 0.004) (Table 2).

Compared with *A. artemisiifolia*, adults of *O. communa* placed on *H. tuberosus* (*z* = 2.78, *p* = 0.005), *B. cernua* (*z* = 4.33, *p* < 0.001), *C. sulphureus* (*z* = 10.64, *p* < 0.001), *P. spinosa* (*z* = 10.94, *p* < 0.001), *P. bifrons* (*z* = 8.64, *p* < 0.001), *P. britannicum* (*z* = 3.12, *p* = 0.002), *A. molinieri* (*z* = 3.56, *p* < 0.001), *M. chamomilla* (*z* = 5.50, *p* < 0.001), *E. sumatrensis* (*z* = 10.18, *p* < 0.001), *B. perennis* (*z* = 13.28, *p* < 0.001), and *C. solstitialis* (*z* = 8.59, *p* < 0.001) had an increased risk of dying (Supplementary Figure S2). There were no statistical differences for the other species. The combined scores and Preference Performance Index can be found in Supplementary Table S5.

When grouping *H. annuus* cultivars together, we found no interaction effect between sex and plant species on pupal weight (*Estimate* = 0.328, *t* = 0.203, *p* = 0.230). Pupae, both males and females, that developed on *H. annuus* had a significantly lower weight (*Estimate* = −0.441, *t* = −3.249, *p* = 0.001, Supplementary Figure S3) compared to the ones obtained from *A. artemisiifolia*.

**Figure 2:**
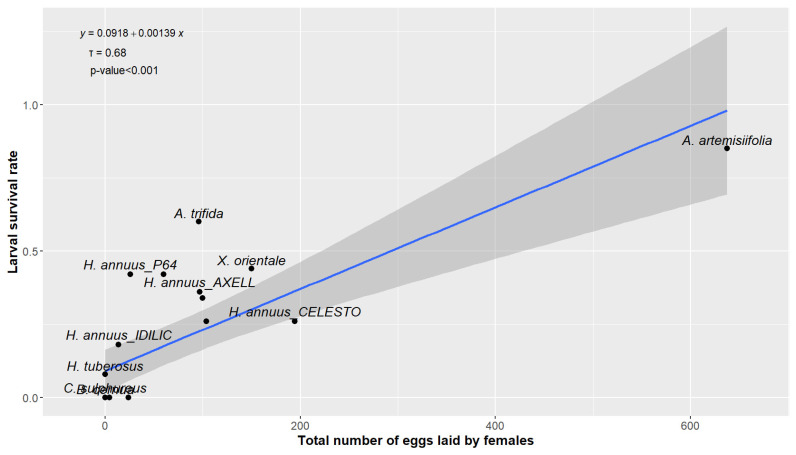
Relationship between larval survival rate (mean) and the total number of eggs laid by *O. communa* females (mean) in no-choice tests. *Tau* corresponds to Kendall’s rank correlation coefficient.

Kendall’s test showed a significant and positive correlation between the number of eggs laid per females and the larval survival (*z* = 3.98; *tau* = 0.6845; *p* < 0.001; Figure 2).

### 3.2. Choice Tests

**Figure 3:**
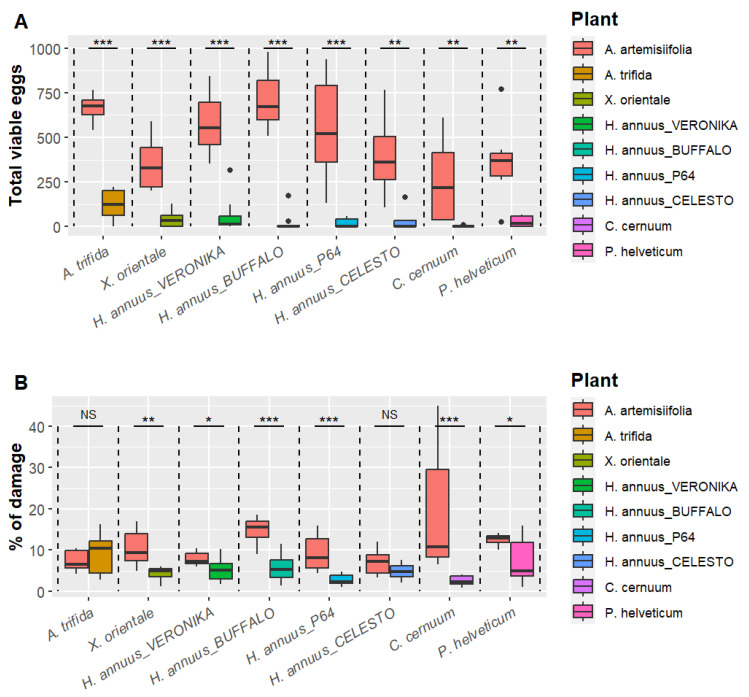
Results of the choice tests performed with *Ophraella communa*: (**A**) total viable eggs laid on each plant and (**B**) herbivory damage after three weeks. The *p*-values were calculated with a Wilcoxon rank sum test with continuity correction. Significance levels: NS non-significant; * *p* < 0.05; ** *p* < 0.01; *** *p* < 0.001.

There were significantly fewer egg masses and viable eggs laid on each non-target plant species tested compared to *A. artemisiifolia* (Figure 3). The non-target plant with the most eggs laid was *A. trifida*, with a mean (SE) of 124 (±84) eggs laid per plant against 667 (±77) on *A. artemisiifolia*. Excluding *C. cernuum* for which only one egg was laid, the mean number of eggs laid on *A. artemisiifolia* was 5.4 to 29.7 times higher than that on non-target species (Supplementary Table S3).

All tested plants showed feeding damage by *O. communa*. At the end of the test (after three weeks), *A. artemisiifolia* was always more damaged than non-target plant species except for *A. trifida* for which the difference was not statistically supported (Figure 3 and supplementary Table S4). While damage to most non-target species increased slightly from one week to the next, feeding damage to *A. artemisiifolia* almost doubled each week.

## 4. Discussion

Our study brings new insights on the host range of *O. communa*, which was poorly documented in the European context. We showed a relative strong specialization of *O. communa* for *A. artemisiifolia*, with its host range restricted to the Heliantheae tribe, and behavioral assessments confirmed the strong preference of female beetles for *A. artemisiifolia*. The results are in line with previous studies on the *O. communa* host range and suggest a low risk for agriculture and the environment.

No-choice tests can provide a good initial screening to determine the fundamental host range of *O. communa* [47,48]. This fundamental host range is defined by all hosts that, given synchronous phenology, are used by a target organism when no alternative is offered—i.e., independent of any environmental setting [48]. However, no-choice tests can sometimes overestimate risk, as the fundamental host range is usually wider than the realized host range (i.e., plant species used under natural conditions) [49,50]. Therefore, to refine our conclusions, we integrated choice tests in our analysis. The combination of these tests revealed that only plants from the Heliantheae tribe sustained larval development to adulthood, while for the Helenieae tribe, limited egg laying can occur. We therefore conclude that *O. communa*’s fundamental host range is restricted to the Heliantheae tribe, which supports the results of previous studies [26,29].

European species are more distantly related to *A. artemisiifolia*. As phylogenetic distance increases between the target species (i.e., *A. artemisiifolia* in our study) and the plants being tested, the risk of non-target attacks generally decreases. However, including more distantly related species is crucial for risk assessment, as it has been shown that host acceptability does not always decline predictably with increasing phylogenetic distance from the target plant [51,52]. In our study, we did not observe such an effect.

The no-choice tests showed some toxic effects of native European plants species on *O. communa* (larvae and adults). However, in the choice tests, *C. cernuum* and *P. helveticum* were consumed by adults, and a few eggs were laid on them, as observed by Augustinus et al., (2020) [26]. These two indigenous species are both from the Inulinae sub-tribe. Plants from this sub-tribe have a well-known negative effect on Coleoptera species. For example, *Dittrichia* (=*Inula*) *graveolens* essential oils have repellent properties against the beetle *Tribolium castaneum* [53], and compound extracts from *Dittrichia viscosa* (*Inula viscosa* L.) have lethal effects on the cowpea seed beetle *Callosobruchus maculatus* (Coleoptera, Chrysomelidae) [54] and on the lesser grain borer *Rhyzopertha dominica* (F.) (Coleoptera, Bostrichidae) [55]. Even though Inulinae species have such toxic effects, as the perception of blends of plant volatiles plays an important role in host recognition [56], we can hypothesize that some Inulinae species share some volatile organic compounds (VOCs) with *A. artemisiifolia* and might thus have been somewhat attractive to *O. communa*. According to Son et al. (2021) [57], the dominant leafy VOCs in *A. artemisiifolia* were spathulenol, caryophyllene oxide, α-curcumene, valencene, borneol, and β-caryophyllene. Caryophyllene oxide and α-curcumene were also found in two Heleniae species [58] as well as several other low-concentrated VOCs. It could be interesting to find out which of these compounds play a role in host recognition by *O. communa* and to compare the attractiveness of Inulinae species with *A. artemisiifolia* in the field. Given the 100% mortality of *O. communa* larvae on every Inulinae species tested, we consider the risk of non-target effects by *O. communa* on these plant species to be very low.

Furthermore, the positive correlation between the number of eggs laid by *O. communa* females on a plant species and the larval survival rate on that plant species supports the “mother know best” hypothesis, also known as the preference–performance hypothesis [59]. This suggests a specialization of *O. communa* for the Heliantheae tribe, particularly for the Ambrosiinae sub-tribe. This is a key feature of *O. communa* for its potential use as a biological control agent, suggesting a reduced likelihood of an evolution of specialization [60] and thus a limited environmental risk because only a few species of Heliantheae are native to Europe.

It should be noted that although the larval survival rate in no-choice conditions and the fecundity parameters in no-choice and choice tests (i.e., number of eggs laid and number of egg masses laid) were always much lower on non-target plants than on *A. artemisiifolia*, some adults still consumed some non-target plants in choice tests, particularly at the beginning of the tests. For two sunflower cultivars (CELESTO and VERONIKA), the feeding damage was slightly higher than that of *A. artemisiifolia* during the first week. However, this damage remained very low (less than 5%, Supplementary Table S7), and as the tests progressed, the insects consumed *A. artemisiifolia* more, and damage to non-target plants did not increase much, except for *A. trifida*—the phylogenetically closest species to *A. artemisiifolia*. This can be explained by the wider host range of adults compared to larvae [26,61] and also by our laboratory conditions that are still far from field conditions. These conditions may cause false positives (i.e., plants may have been chosen in the laboratory but not be part of the realized host range) [48].

Moreover, considering the correlation between relative performance and risk of non-target attack, showed by Paynter et al. (2015) and Grevstad et al. (2021) [45,46], we calculated the “combined score” of our results. All combined scores are well below the threshold (0.33) found by Paynter et al. (2015). However, the Preference and Performance Index (PPI) identified by Grevstad et al. (2021) is more controversial, as they did not find a clear threshold, but the lowest data point value of the PPI with positive full field use was 0.226 [46], and most of our PPI scores are below that number. Three species, however, presented contrasting results. For *A. trifida*, the PPI score was higher (0.374), suggesting a potential risk of non-target impact. While it has been documented that *O. communa* can feed on *A. trifida* in Asia [62], it has not been reported in Europe. Any non-target impact on *A. trifida* might be viewed as beneficial, as it is also an invasive species in France and is considered a major agricultural and public health threat. Two other species have PPI scores close to 0.226: *Xanthium orientale* (0.258) and the sunflower cultivar VERONIKA (0.250). Although the PPI indicates a low risk of non-target effects on these species, we should be cautious in interpreting these findings. First, the observed values are very close to the threshold. Second, the first species, *X. orientale*, is also a harmful invasive weed in France and Europe, meaning any impact on this species could be considered beneficial. The second case involves *Helianthus annuus*, which is an important crop.

Because *H. annuus* is part of the Heliantheae tribe and is an important crop in France, we included several sunflower cultivars used in the country in our study. Seven cultivars were selected, based on distinct characteristics such as oil or seed production and their respective maturation periods, to screen the diversity of sunflowers cultivated in France. The results revealed a low number of eggs laid on these various cultivars, with minimal leaf consumption by the adults. Furthermore, larval survival rates were 2.7 times lower than those observed on *A. artemisiifolia* (with an average survival rate of 0.85 on *A. artemisiifolia* compared to 0.32 on *H. annuus*). Similarly, the pupal weights of individuals that had developed on *A. artemisiifolia* were significantly greater than those that had developed on *H. annuus*. Pupal weight may serve as a proxy for adult fitness, particularly as it can have an impact on individual fecundity [63] or their ability to survive the cold (fewer accumulated lipid reserves). For instance, research on *Helicoverpa armigera* (Hübner) (Lepidoptera: Noctuidae) has highlighted how host plant species influence pupal size, affecting insects’ entry into diapause [64]. Although the beetle was rejected for introduction in Australia as a biological control agent due to its ability to complete its life cycle on *H. annuus* under laboratory conditions [29], which was confirmed by our study, several international studies have, in addition, observed the behavior of *O. communa* in outdoor experiments with sunflowers, and all had the same conclusion: *O. communa* poses no threat to *H. annuus* yield [27,29,31,65]. Moreover, the tested cultivars are either sold by Italian companies or available in Italy, and since its accidental introduction in Italy and Switzerland more than ten years ago, only very minor and rare damage has been reported on sunflowers. Finally, as adult survival was not significantly affected by *H. annuus*, conducting multigeneration host specificity testing (or continuation trials) could provide a more precise assessment of the risk of adaption to *H. annuus*. This is currently being evaluated. However, a field cage study conducted in Italy showed that *O. communa* populations rapidly declined when continuously reared on sunflowers, with no beetles found after three years [15]. Given this, the risk of *O. communa* causing significant impact to sunflower production in France seems low and/or negligible.

Our study showed that larval survival on *A. trifida* was higher than all other test plants (except for *A. artemisiifolia*). Fukano et al. (2016) reported that *O. communa* does not use *Ambrosia trifida* as a host plant in North America but that the beetle actively feeds on this plant in its introduced range in Japan [62]. Attacks on closely related plant species are not unusual; indeed, most non-target attacks in weed biocontrol programs occur on plants that are congeneric with the target weed [66]. Fukano et al. (2016) also found that Japanese populations of *O. communa* perform better on *A. trifida* than their North American counterparts, suggesting that this expansion likely occurred because Japanese *A. trifida* populations are less resistant to *O. communa* herbivory than North American populations [62]. To our knowledge, *O. communa* has not been reported on *A. trifida* in Europe, and in our study, we did not observe an increased performance of *O. communa* on *A. trifida*. However, our results suggest that *A. trifida* could potentially become a host for the beetle in France. Any non-target impact on *A. trifida* might be viewed as beneficial, as it is also an invasive species in France and is considered a major agricultural and public health threat [67].

Given the recent discovery of *O. communa* in France, there is an urgent need to complete host range risk analyses to non-target plant species, as suggested by the French Agency for Food, Environmental and Occupational Health & Safety (ANSES) in 2015 and 2019 [21,68]. The centrifugal phylogenetic method used in this study to establish the list of plant species to be tested is well recognized to evaluate the host range of biological control agents [24,25,69,70]. Therefore, the plants we selected combined with the plant species already tested by Augustinus et al. (2020) should cover a sufficiently wide range of tribes to safely evaluate the risk for non-target species [26]. More species from the Heliantheae or Helenieae tribes could have been tested, but this was not achieved during our study owing to logistic constraints as well as the very low (to null) survival of *O. communa* larvae on plants belonging to these tribes; in this instance, the present results are able to predict no risk to these plant species.

Our results indicate no risk of significant non-target effects by *O. communa* to European indigenous plant species and low risk to introduced plant species. Given the ten-year history of this species in Italy and eastern Europe and our results, the use of *O. communa* as a biological control agent against *A. artemisiifolia* looks very promising. Nevertheless, we suggest monitoring the spread of the recently introduced *O. communa* in France and its potential impact on non-target plant species in Europe.

## Figures and Tables

**Table 1:** Plant species used for host specificity testing of *Ophraella communa*. Plants species are ordered by their phylogenetic relatedness to *Ambrosia artemisiifolia*.

Tribe	Sub-Tribe	Species	Cultivars	INPN Status		No-Choice Test	Choice Test
Heliantheae	Ambrosiinae	*Ambrosia artemisiifolia* L.		Introduced	I	x	x
		*Ambrosia trifida* L.		Introduced	NE	x	x
		*Xanthium orientale* L.		Introduced	NE	x	x
	Helianthinae	*Helianthus annuus* L.	ES VERONIKA	Cultivated	LC	x	x
			RGT BUFFALO	Cultivated	LC	x	x
			P64HE118	Cultivated	LC	x	x
			SY CELESTO	Cultivated	LC	x	x
			RGT AXELL	Cultivated	LC	x	
			MAS 89HOCL	Cultivated	LC	x	
			ES IDILIC	Cultivated	LC	x	
		*Helianthus tuberosus* L.		Cultivated	I	x	
	Coreopsidinae	*Bidens cernua* L.		Indigenous	LC *	x	
		*Cosmos sulphureus* Cav.		Cultivated	NE	x	
Helenieae	Inulinae: Pulicaria complex	*Pallenis spinosa* L.		Indigenous	LC **	x	
	Inulinae: Inula complex	*Carpesium cernuum* L.		Indigenous	EN	x	x
		*Pentanema bifrons* L.		Indigenous	LC **	x	
		*Pentanema britannicum* L.		Indigenous	NT **	x	
		*Pentanema helveticum* (Weber)		Indigenous	LC **	x	x
Anthemideae	Anthemidinae	*Artemisia molinieri* Quézel.		Endemic	EN	x	
		*Matricaria chamomilla* L.		Indigenous	LC	x	
Astereae	Conyzinae	*Erigeron sumatrensis* Retz.		Introduced	NE	x	
	Asterinae	*Bellis perennis* L.		Indigenous	LC	x	
Carduoideae	Centaureinae	*Centaurea solstitialis* L.		Indigenous	LC **	x	

INPN status corresponded to indicators on the conservation status of each species in France. These indicators are provided by Inventaire National du Patrimoine Naturel: I = invasive; NE = Not evaluated; LC = least concern; NT = Near threatened; EN = in danger; * = NT in some regions in France; ** = EN in some regions in France [36].

**Table 2:** No-choice results: preoviposition period, larval development time, pupal weight, and sex ratio (percent of females). When possible (more than two replicates), *p*-values were calculated using the Benjamin and Hochbeg (1995) method in a Wilcoxon test. ^a^ Number of plants tested with observed oviposition (five replicates per species, *A. artemisiifolia* was used as control in each session). ^b^ Number of larvae that survived until adulthood (10 larvae per replicate, five replicate per species, *A. artemisiifolia* was used as control in each session). NS: non-significant; *: *p* < 0.1; **: *p* < 0.01.

Species	Cultivars	Preoviposition Period			Larval Development Time			Pupal Weight		Sex Ratio (Female)
		^a^	Average	p.adj	^b^	Number of Days	p.adj	Female	Male	Average (%)
*Ambrosia artemisiifolia* L.		32	7.3 ± 1.5	-	237	24.96 ± 2.1	-	7.77 ± 1.31	5.93 ± 0.98	49.26 ± 16.4
*Ambrosia trifida* L.		5	9.8 ± 3.1	0.037 *	30	24.30 ± 1.9	0.112 NS	7.05 ± 1.29	5.91 ± 1.42	57.86 ± 10.2
*Xanthium orientale* L.		5	8.6 ± 0.9	0.04 *	22	24.23 ± 1.7	0.290 NS	7.74 ± 1.07	5.44 ± 0.97	56.67 ± 26.2
*Helianthus annuus* L.										
	ES VERONIKA	5	13.6 ± 5.0	0.031 *	21	25.00 ± 2.7	0.896 NS	7.29 ± 1.00	6.60 ± 1.18	53.00 ± 13.0
	RGT BUFFALO	5	9.8 ± 1.8	0.026 *	16	25.62 ± 3.6	0.896 NS	6.54 ± 0.59	5.04 ± 0.84	46.67 ± 36.1
	P64HE118	3	11.0 ± 3.6	0.067 NS	20	25.16 ± 2.7	0.896 NS	7.94 ± 1.41	5.89 ± 0.98	53.75 ± 36.7
	SY CELESTO	4	9.5 ± 2.9	0.124 NS	12	23.08 ± 1.4	0.005 **	6.40 ± 0.35	5.22 ± 0.30	56.67 ± 43.5
	RGT AXELL	5	15.2 ± 4.4	0.003 **	17	23.94 ± 2.5	0.189 NS	8.30 ± 0.83	5.57 ± 0.51	38.67 ± 23.6
	MAS 89HOCL	5	10.6 ± 3.3	0.04 *	11	27.27 ± 1.7	0.003 **	6.89 ± 1.28	5.60 ± 0.99	85.41 ± 17.2
	ES IDILIC	2	15.0 ± 8.5	-	9	25.22 ± 1.1	0.744 NS	6.23 ± 0.61	5.12 ± 0.51	36.11 ± 37.6
*Helianthus tuberosus* L.		-	-	-	4	30.25 ± 2.9	0.005 **	5.63 ± 1.75	4.60	75.00 ± 35.4
*Carpesium cernuum* L.		4	16.5 ± 1.0	0.004 **		-	-	-	-	-
*Pentanema helveticum* (Weber)		2	15.0 ± 7.1	-		-	-	-	-	-

## Data Availability

The data presented in this study are available on reasonable request from the corresponding author.

## Supplementary Materials

The following supporting information can be downloaded at https://www.mdpi.com/article/10.3390/plants13223240/s1. Reference [71] is cited in the supplementary materials.
