# Supplementary material for: Assessing the Host Range of Ophraella communa for the Biological Control of Ambrosia artemisiifolia in France
Source: Plants (Basel). 2024 Nov 19;13(22):3240. doi: 10.3390/plants13223240 (PMC11598483; doi:10.3390/plants13223240)
Supplement: Supplementary file 1 [file plants-13-03240-s001.zip › plants-3244149-supplementary.pdf]

Supplementary material

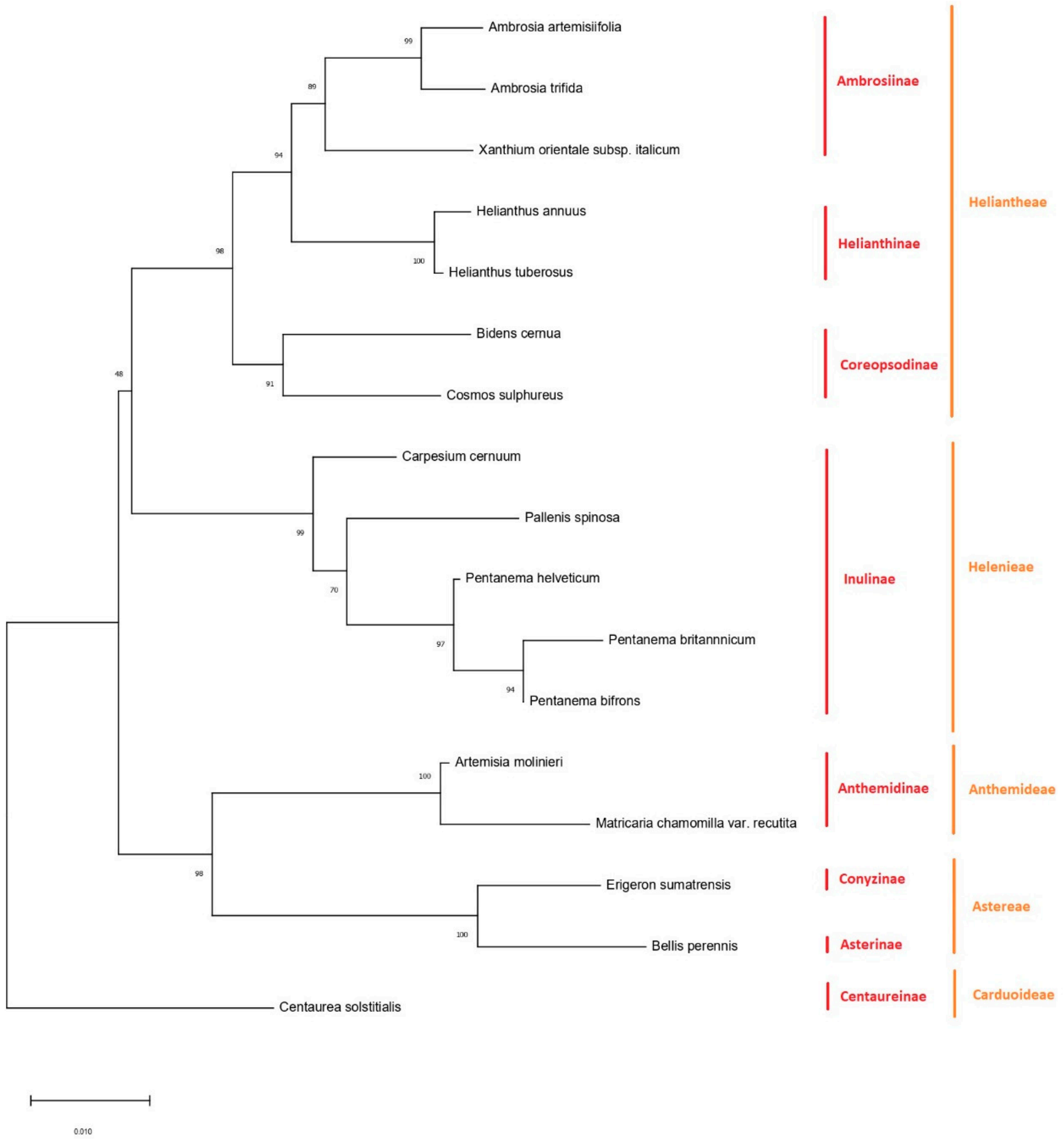

Supplementary material: Figure S1. Phylogenetic tree of the tested plants. Orange text is the plant species tribe, and red text is the sub-tribe. The genetic distances were obtained by analysing the sequences of a portion of the matK (maturase K) gene. The sequences for each plant species were downloaded from the GenBank database (https ://www.ncbi.nlm.nih.gov/genbank/). The sequences were then aligned using the Clustal W method. Genetic distances are represented by the number of base substitutions per site between sequences. Analyses were performed using the 3-parameter Tamura model. Rate variation between sites was modelled using a gamma distribution. The final dataset comprised a total of 440 positions. All analyses were performed via MEGA X software [71].

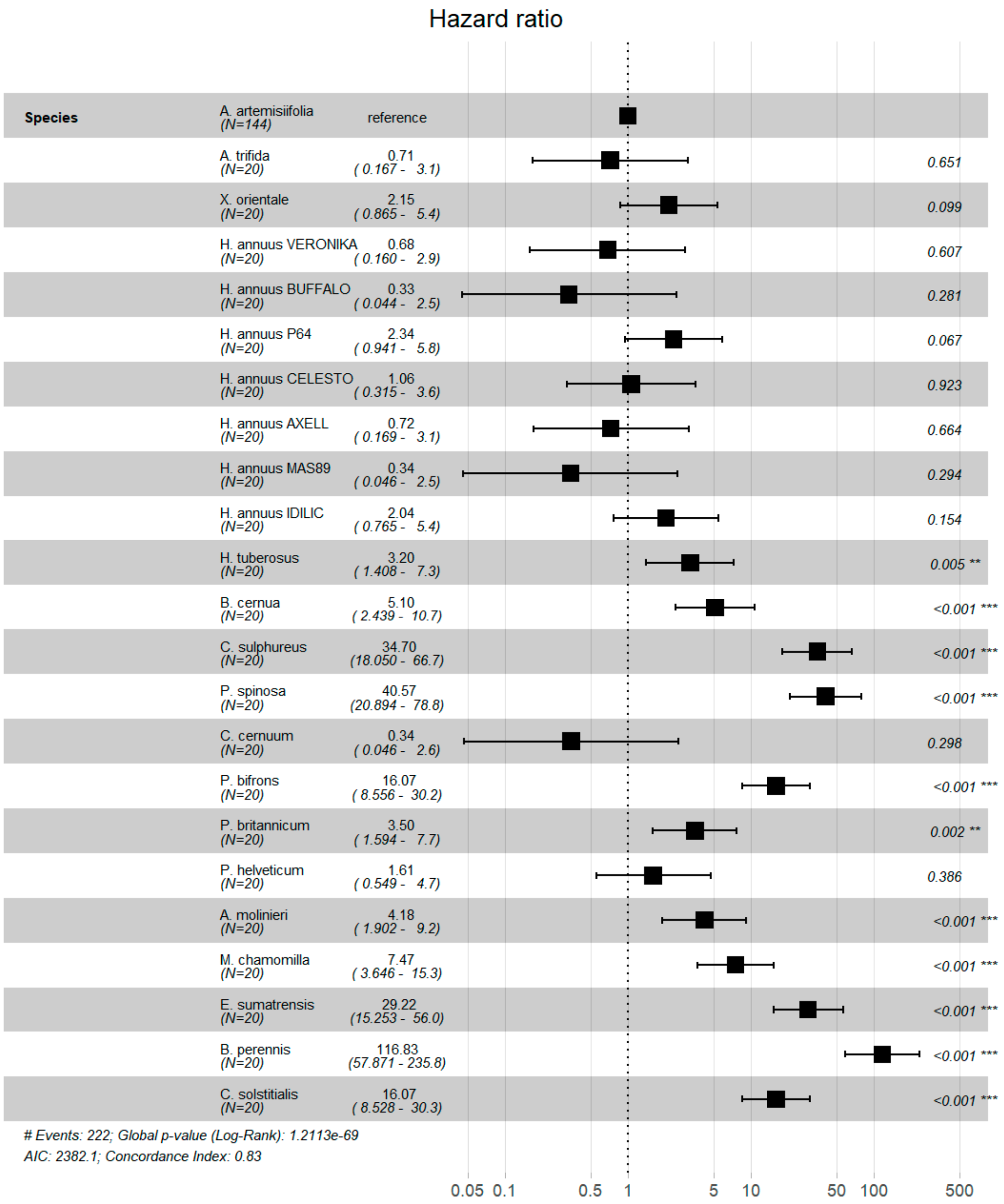

Supplementary material: Figure S2. Estimated effect sizes +/- 95CI for the difference in adult survival between the test plant and *A. artemisiifolia* (forest plot based on a Cox proportional hazards model adjusted for adult survival data). When the hazard ratio is greater than 1, it indicates an increase in the mortality risk compared with *A. artemisiifolia*, whereas if it is less than 1, it indicates a decrease in the mortality risk compared with *A. artemisiifolia*.

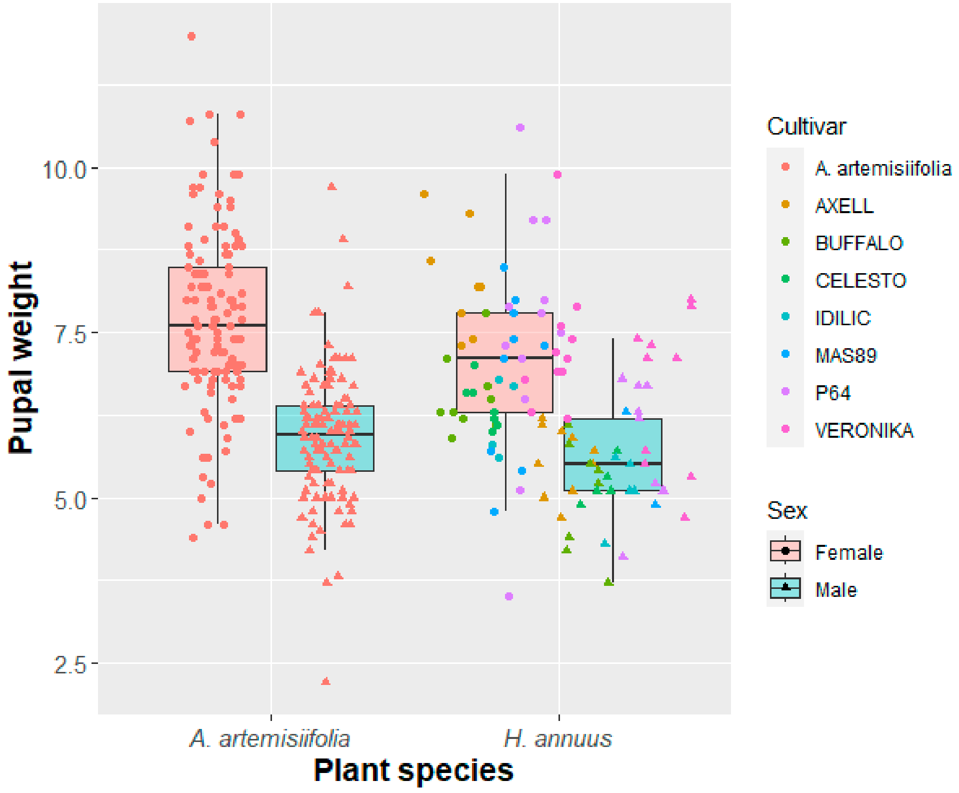

Supplementary material: Figure S3. Pupal weight depending on the sex of the individuals and the plant tested (*Artemisia artemisiifolia* and *Helianthus annuus*) in no-choice test. Female individuals are represented by circles (reddish boxplot), and males by triangle (blue boxplot)

Supplementary material: Table S1. Origins of seeds used in our experiments.

Species	Cultivars	Seeds origin
Ambrosia artemisiifolia L.		Côte d'Or (France)
Ambrosia trifida L.		Ariège (France)
Xanthium orientale L.		Hérault (France)
Helianthus annuus L.	ES VERONIKA	LIDEA
	RGT BUFFALO	RAGT Semences
	P64HE118	Pioneer Semences
	SY CELESTO	Syngenta
	RGT AXELL	RAGT Semences
	MAS 89HOCL	Mas Seeds
	ES IDILIC	LIDEA
Helianthus tuberosus L.		Gard (France)
Bidens cernua L.		MNHN
Cosmos sulphureus Cav.		Hérault (France)
Pallenis spinosa L.		Hérault (France)
Carpesium cernuum L.		Geneva Botanical Gardens
Pentanema bifrons L.		Botanical Garden La Gacilly (Brittany, France)
Pentanema britannicum L.		NBC Brest (France)
Pentanema helveticum (Weber)		Geneva Botanical Gardens
Artemisia molinieri Quézel.		NBC Brest (France)
Matricaria chamomilla L.		Hal (Belgium)
Erigeron sumatrensis Retz.		Hérault (France)
Bellis perennis L.		Hal (Belgium)
Centaurea solstitialis L.		Hérault (France)

Supplementary material: Table S2: No-choice results: Egg masses, total of viable eggs and larva survival rate. P-values were calculated with Benjamin & Hochbeg (1995) method in a Wilcoxon test. [a] Number of replicates used in the larvae test.

Sub-tribe	Species	Replicate	Egg batches		Total of viable eggs		Larval survival		
			Average	p-value	Average	p-value	a	Average	p-value
Ambrosiinae	*Ambrosia artemisiifolia* L.	32	26.7 ± 11.7	-	637.1 ± 374.1	-	26	0.85 ± 0.1	-
	Ambrosia trifida L.	5	10.2 ± 10.2	0.011 *	96.0 ± 110.0	<0.001 ***	5	0.60 ± 0.2	<0.001 **
	Xanthium orientale L.	5	10.4 ± 5.00	0.003 **	150.2 ± 119.5	0.002 **	5	0.44 ± 0.2	<0.001 ***
Helianthinae	*Helianthus annuus* L.								
	ES VERONIKA	5	5.8 ± 5.17	0.002 **	60.0 ± 70.7	<0.001 ***	5	0.42 ± 0.2	0.002 **
	RGT BUFFALO	5	7.2 ± 4.76	0.003 **	99.8 ± 87.0	<0.001 ***	5	0.34 ± 0.1	<0.001 ***
	P64HE118	5	2.4 ± 2.61	<0.001 ***	25.8 ± 30.2	<0.001 ***	5	0.42 ± 0.2	<0.001 ***
	SY CELESTO	5	11.4 ± 4.51	0.006 **	194.4 ± 113.5	0.006 **	5	0.26 ± 0.1	<0.001 ***
	RGT AXELL	5	5.2 ± 6.87	0.002 **	96.8 ± 156.2	0.002 **	5	0.36 ± 0.2	<0.001 ***
	MAS 89HOCL	5	6.0 ± 7.04	0.002 **	103.8 ± 149.5	<0.001 ***	5	0.26 ± 0.1	<0.001 ***
	ES IDILIC	5	1.4 ± 2.61	<0.001 ***	13.8 ± 28.7	<0.001 ***	5	0.18 ± 0.2	<0.001 ***
	Helianthus tuberosus L.	5	0	<0.001 ***	0	<0.001 ***	5	0.08 ± 0.1	<0.001 ***
Coreopsidinae	*Bidens cernua* L.	5	0	<0.001 ***	0	<0.001 ***	5	0	<0.001 ***
	Cosmos sulphureus Cav.	5	0	<0.001 ***	0	<0.001 ***	5	0	<0.001 ***
Inulinae : Pulicaria complex	*Pallenis spinosa* L.	5	0	<0.001 ***	0	<0.001 ***	5	0	<0.001 ***
Inulinae : Inula complex	*Carpesium cernuum* L.	5	3.0 ± 2.35	<0.001 ***	24.0 ± 23.0	<0.001 ***	5	0	<0.001 ***
	Pentanema bifrons L.	5	0	<0.001 ***	0	<0.001 ***	5	0	<0.001 ***
	Pentanema britannicum L.	5	0	<0.001 ***	0	<0.001 ***	5	0	<0.001 ***
	Pentanema helveticum (Weber)	5	0.8 ± 1.30	<0.001 ***	4.4 ± 6.0	<0.001 ***	5	0	<0.001 ***
Anthemidinae	*Artemisia molinieri* Quézel.	5	0	<0.001 ***	0	<0.001 ***	5	0	<0.001 ***
	Matricaria chamomilla L.	5	0	<0.001 ***	0	<0.001 ***	5	0	<0.001 ***
Conyzinae	*Erigeron sumatrensis* Retz.	5	0	<0.001 ***	0	<0.001 ***	5	0	<0.001 ***
Asterinae	*Bellis perennis* L.	5	0	<0.001 ***	0	<0.001 ***	5	0	<0.001 ***
Centaureinae	*Centaurea solstitialis* L.	5	0	<0.001 ***	0	<0.001 ***	5	0	<0.001 ***

Supplementary material: Table S3. Choice test results: egg masses and total viable eggs on each plant. The p-value were calculated with a Wilcoxon rank sum test with continuity correction between A. artemisiifolia and the non-target plant. For each non-target plant species, the data on A. artemisiifolia and the plant tested are side-by-side, as they correspond of the same test.

Species	Cultivars	Replicate	Egg masses on each plant						Total viable eggs on each plant					
			A. artemisiifolia		Non-target		p-value		*A. artemisiifolia*		Non-target		p-value	
Ambrosia trifida L.		8	23.6	± 2.1	5.1	± 3.3	<0.001	***	666.5	± 76.9	124.1	± 83.5	<0.001	***
Xanthium orientale L.		8	18.3	± 6.0	2.6	± 3.2	0.0013	**	350.9	± 142.8	41.0	± 46.3	<0.001	***
Helianthus annuus L.														
	ES VERONIKA	8	26.4	± 5.4	3.0	± 3.9	<0.001	***	606.9	± 186.1	69.3	± 109.2	<0.001	***
	RGT BUFFALO	8	28.8	± 6.3	1.0	± 2.5	<0.001	***	708.8	± 172.3	25.5	± 61.3	<0.001	***
	P64HE118	8	27.5	± 9.1	0.8	± 0.9	<0.001	***	550.1	± 285.9	18.5	± 26.1	<0.001	***
	SY CELESTO	8	19.5	± 8.2	1.4	± 2.4	<0.001	***	405.1	± 229.1	29.3	± 56.2	0.0013	**
Carpesium cernuum L.		7	13.0	± 9.3	0.1	± 0.4	0.0014	**	253.9	± 247.4	1.1	± 3.0	0.0014	**
Pentanema helveticum (Weber)		7	18.1	± 8.7	1.9	± 2.4	0.0038	**	364.9	± 223.4	29.1	± 31.6	0.0070	**

Supplementary material: Table S4. Choice test results: herbivory damages on each plant after one, two and three weeks. The p-value were calculated with a Wilcoxon rank sum test with continuity correction. For each non-target plant species, the data on *A. artemisiifolia* and the plant tested are side-by-side, as they correspond of the same test.

Species	Herbivory after one week						Herbivory after two weeks						Herbivory after three weeks					
	A. artemisiifolia		Non-target		p-value		*A. artemisiifolia*		Non-target		p-value		*A. artemisiifolia*		Non-target		p-value	
Ambrosia trifida L.	2.90	± 1.73	3.88	± 4.02	1	NS	6.52	± 4.60	9.32	± 5.80	0.38	NS	8.49	± 5.10	9.08	± 4.92	0.8785	NS
Xanthium orientale L.	3.39	± 2.25	3.18	± 1.73	1	NS	5.75	± 3.13	3.29	± 1.68	0.1049	NS	10.48	± 4.52	4.61	± 2.28	0.0047	**
Helianthus annuus L.																		
Veronika	1.37	± 0.83	4.01	± 3.20	0.0070	**	4.80	± 2.38	4.79	± 3.38	0.6454	NS	7.88	± 1.71	5.32	± 3.19	0.0499	*
Buffalo	5.22	± 2.46	3.65	± 1.60	0.1605	NS	10.81	± 4.08	4.70	± 1.93	0.0030	**	14.79	± 3.22	5.70	± 3.54	<0.001	***
P64	2.71	± 1.50	3.08	± 2.88	0.6454	NS	4.81	± 2.41	5.23	± 5.36	0.7209	NS	9.17	± 4.27	2.79	± 1.32	<0.001	***
Celesto	1.18	± 0.94	3.49	± 1.30	0.0011	**	3.54	± 1.70	4.33	± 1.43	0.4418	NS	7.08	± 3.09	4.87	± 1.97	0.1605	NS
Carpesium cernuum L.	5.27	± 4.22	2.80	± 2.46	0.1807	NS	10.97	± 8.81	2.34	± 1.28	0.0070	**	19.68	± 15.18	2.90	± 1.89	<0.001	***
Pentanema helveticum (Weber)	1.92	± 0.99	2.20	± 1.55	0.62	NS	7.20	± 1.16	5.97	± 5.65	0.1649	NS	12.50	± 1.41	7.57	± 5.64	0.0973	*

Supplementary material: Table S5. Relative performance combined score, and Preference and Performance Index (PPI) calculated as describe by Paynter et al. (2015) and Grevstad et al. (2021)

Plant species	Cultivars	Relative performance		Combined score	PPI
		Oviposition	Larval survival		
A. trifida		0.151	0.703	0.106	0.374
X. orientale		0.236	0.515	0.121	0.258
H. annuus	SY CELESTO	0.305	0.305	0.093	0.161
	RGT BUFFALO	0.157	0.398	0.062	0.137
	ES VERONIKA	0.094	0.492	0.046	0.250
	P64HE118	0.040	0.492	0.020	0.148
C. cernuum		0.038	0.000	0.000	0.012
P. helveticum		0.007	0.000	0.000	0.030
